# Production and Comprehension of Gestures between Orang-Utans (*Pongo pygmaeus*) in a Referential Communication Game

**DOI:** 10.1371/journal.pone.0129726

**Published:** 2015-06-19

**Authors:** Richard Moore, Josep Call, Michael Tomasello

**Affiliations:** 1 Department of Developmental and Comparative Psychology, Max Planck Institute for Evolutionary Anthropology, Leipzig, Saxony, Germany; 2 Berlin School of Mind and Brain, Humboldt-Universität zu Berlin, Berlin, Germany; 3 School of Psychology and Neuroscience, University of St Andrews, St Andrews, Fife, Scotland; University of Portsmouth, UNITED KINGDOM

## Abstract

Orang-utans played a communication game in two studies testing their ability to produce and comprehend requestive pointing. While the ‘communicator’ could see but not obtain hidden food, the ‘donor’ could release the food to the communicator, but could not see its location for herself. They could coordinate successfully if the communicator pointed to the food, and if the donor comprehended his communicative goal and responded pro-socially. In Study 1, one orang-utan pointed regularly and accurately for peers. However, they responded only rarely. In Study 2, a human experimenter played the communicator’s role in three conditions, testing the apes’ comprehension of points of different heights and different degrees of ostension. There was no effect of condition. However, across conditions one donor performed well individually, and as a group orang-utans’ comprehension performance tended towards significance. We explain this on the grounds that comprehension required inferences that they found difficult – but not impossible. The finding has valuable implications for our thinking about the development of pointing in phylogeny.

## Introduction

Pointing is a form of intentional, referential communication that has been hypothesised to play a foundational role in human communicative and cognitive development [1]. By enabling communicators to coordinate their behaviour with respect to a distal feature of the environment, it can facilitate numerous activities that are central to the development of human culture–including language acquisition and pedagogy. It was likely also fundamental in enabling our early hominin ancestors to engage in the large game hunting that supported an increasingly carnivorous diet and which, in turn, supported further cognitive growth [2]. However, pointing is widely recognised to be cognitively challenging [1] [3]. For coordination to be possible, the recipient of a point must be able to make potentially difficult inferences about her interlocutor’s communicative goal–both identifying the referent of the point (the ‘referential intention’), and goal the with which the communicator sought to direct that referent to her attention (the ‘social intention’). For this reason, it is often argued that the cognitive abilities that enabled pointing production and comprehension in humans are not widely shared by other species [1] [4].

A robust finding has been that children as young as twelve months excel at both producing and comprehending points [5] [6] [7]. However, while captive apes (those living in zoos and sanctuaries but not hand-reared by humans) sometimes start to point in interaction with humans, their pointing comprehension–where this is operationalised just as an ability to respond appropriately to others’ points (for example, by using an experimenter’s point to locate hidden food)–is comparatively poor [8] [9] [10]. Furthermore, there is little evidence that apes point for one another in the wild (although see [11] [12] [13]).

In interactions with humans, captive apes have been shown to point to make requests–for example, for food, or for tools that will enable them to get food [14] [15] [16]. However, the motivations with which they point are characteristically different from human points. While children point both for the benefit of others and as a means of making requests, apes point only rarely when they do not stand to gain themselves [17]. Furthermore, while infants of 12 months point both distally and for objects that they could acquire themselves, apes tend to point only for objects that they cannot obtain, and only after approaching them [18].

With respect to comprehension, apes typically fare unspectacularly. While children as young as 12 months [5] and even domestic dogs [19] are reliably able to use an experimenter's point to locate hidden food, chimpanzees regularly succeed in this task only at chance [8] [9] [10]–despite being highly motivated. While fewer studies have been conducted on apes’ comprehension of requestive points, chimpanzees have been shown to perform no better than chance when a human experimenter pointed to request from them one of two random objects [20], and all species of non-human great ape performed poorly in a task testing their comprehension of a third party’s requestive point [21].

While some chimpanzees, bonobos, and orang-utans have been shown to perform well in tasks testing their comprehension of informative pointing [14] [22], individuals in these studies were often hand-reared by humans, and so exposed to humans–and human forms of communication–in ways that their peers were not. Moreover, in these studies (e.g., [22]) points were often produced much closer to the targets than in studies with more negative findings. These results suggest that with an upbringing not typical of zoo apes, and a slightly easier experimental setup, apes can be brought to comprehend the points of human interlocutors. This may be because ontogeny plays a foundational role in the development of pointing comprehension abilities in both human and ape subjects, such that when apes are reared in conditions comparable to human children, they acquire skills needed for comprehension that they would otherwise lack [23]. Yet even in such cases of enculturation, there is little evidence of apes spontaneously incorporating pointing gestures into their interactions with one another. In this respect, they differ from human children, who have been observed to point for one another at 12-months [24].

Against this background, a valuable finding was made by Pelé et al. [25], when they reported the spontaneous use of a pointing gesture between conspecifics in populations of captive chimpanzees, bonobos, and orang-utans at Leipzig zoo. In a token-exchange task all species of great ape were trained to exchange different sets of tokens for food. Pairs of conspecifics were then each given a set of tokens containing some that were valuable to themselves, some that were valuable to a partner, and some that were valuable to neither; and they were given the opportunity to exchange tokens with one another. During exchanges, chimpanzees, bonobos, and orang-utans (but not gorillas) all spontaneously used points as a means of requesting from their conspecifics the tokens that they desired. One individual in particular—Bimbo, a nursery-reared adult flanged male orang-utan–was observed to point for other orang-utans frequently. Furthermore, his conspecifics responded appropriately to these points. When Bimbo produced ambiguous begging gestures, he received the tokens he required only at chance levels. By contrast, when he pointed to the tokens that he needed, he received them significantly more often than would be predicted by chance.

While this finding is highly suggestive, a weakness of the used methodology limits what we can infer about the strategies that the orang-utans in this study used to interpret the communicative goals with which Bimbo had pointed. In particular, all females may have had prior knowledge of what it was that Bimbo wanted, because they had previously seen him exchanging the tokens to which he was pointing for food. This suggests a way in which they might perform well on the task without really understanding that Bimbo wanted the object to which he pointed (that is, without grasping the referential intention of his point). One possibility consistent with this is that females did not, in fact, differentiate at all between the pointing and begging gestures Bimbo produced. In both conditions they grasped that he wanted something without knowing what he wanted. However, when Bimbo’s points directed their attention to a subset of tokens, the females may simply have been reminded of his pre-existing preference. This prior knowledge would then give them reason to hand these tokens over. By contrast, when the begging gestures failed to direct their attention to any subset of tokens, they were not reminded of his pre-existing desire; and the females just guessed at what he might want. Were this the case, then Bimbo’s interlocutors would not have inferred the content of his request by grasping that his point indicated that tokens to which he pointed. Rather, they responded appropriately because they already knew what he wanted. Had they been ignorant of what he wanted, such that they could not infer his desire from seeing the object that he desired, they may not have been so successful in interpreting his points.

To test the flexibility of the same orang-utans’ comprehension of pointing–and so to better understand the sorts of inference that they can make in communicative interaction–we tested them again in a similar but more challenging paradigm, in which they could not see the objects that Bimbo wanted.

In order to better evaluate whether orang-utan peers were able to comprehend (that is, respond appropriately to) his points, even in the absence of prior knowledge, we designed a simple coordination game to be played between two individuals. A pair of studies was conducted. In the first, Bimbo was observed interacting with each of five female orang-utans. In the second, Bimbo and each of these females played the same game with a human experimenter.

## Study 1

### Materials and Methods

#### Participants

Participants in Study 1 were six orang-utans (*Pongo pygmaeus*) housed at the Wolfgang Köhler Primate Research Centre at Leipzig zoo: Bimbo (32yrs), a flanged male; four adult females: Pini (23yrs), Padana (14yrs), Kila (11yrs), and Dokana (22yrs); and a sub-adult female (Raaja, 8yrs). Each of the adult females was tested in the company of a dependent offspring who was too young to be separated from them. All subjects participated voluntarily, and were rewarded for their participation with food. The food (a mixture of grapes, banana, and dried banana pellets) was delivered on schedule, and in amounts determined in accordance with their planned daily diet and approved by the zookeepers. Water was available ad libitum.

#### Ethics statement

In accordance with the recommendations of the Weatherall report ‘The Use of Non-Human Primates in Research’ groups of apes were housed in semi-natural indoor (230m^2^) and outdoor (1680m^2^) enclosures containing climbing structures, such as ropes and platforms; and natural features, such as vegetation, trees and streams. Inside these enclosures apes are fed daily at regular intervals, and eat a balanced diet consisting of fruit, vegetables, and plants, supplemented with small amounts of dairy, meat, and carbohydrates. The apes also have access to enrichment devices including shaking boxes and poking bins, and are provided with a further variety of difficult to open food packages through the day. They have access to drinking water ad libitum. Subjects participated voluntarily in the study and were never food or water deprived. Research was conducted in a dedicated observation room (4.65m x 5m x 3.2m) divided into a number of smaller testing facilities (see procedure for precise measurements of the rooms used).

No medical, toxicological or neurobiological research of any kind is conducted at the WKPRC. Research was non-invasive and strictly adhered to the legal requirements of Germany. The study was ethically approved by an internal MPI-EVA committee, consisting of zookeepers and academic research staff. Animal husbandry and research comply with the ‘EAZA Minimum Standards for the Accommodation and Care of Animals in Zoos and Aquaria’, the ‘WAZA Ethical Guidelines for the Conduct of Research on Animals by Zoos and Aquariums’ and the ‘Guidelines for the Treatment of Animals in Behavioral Research and Teaching’ of the Association for the Study of Animal Behavior (ASAB). IRB approval was not necessary because Germany requires no special permission for the use of animals in purely behavioural or observational studies (TierSchGes §7 and §8).

#### Equipment and setup

**Fig 1:**
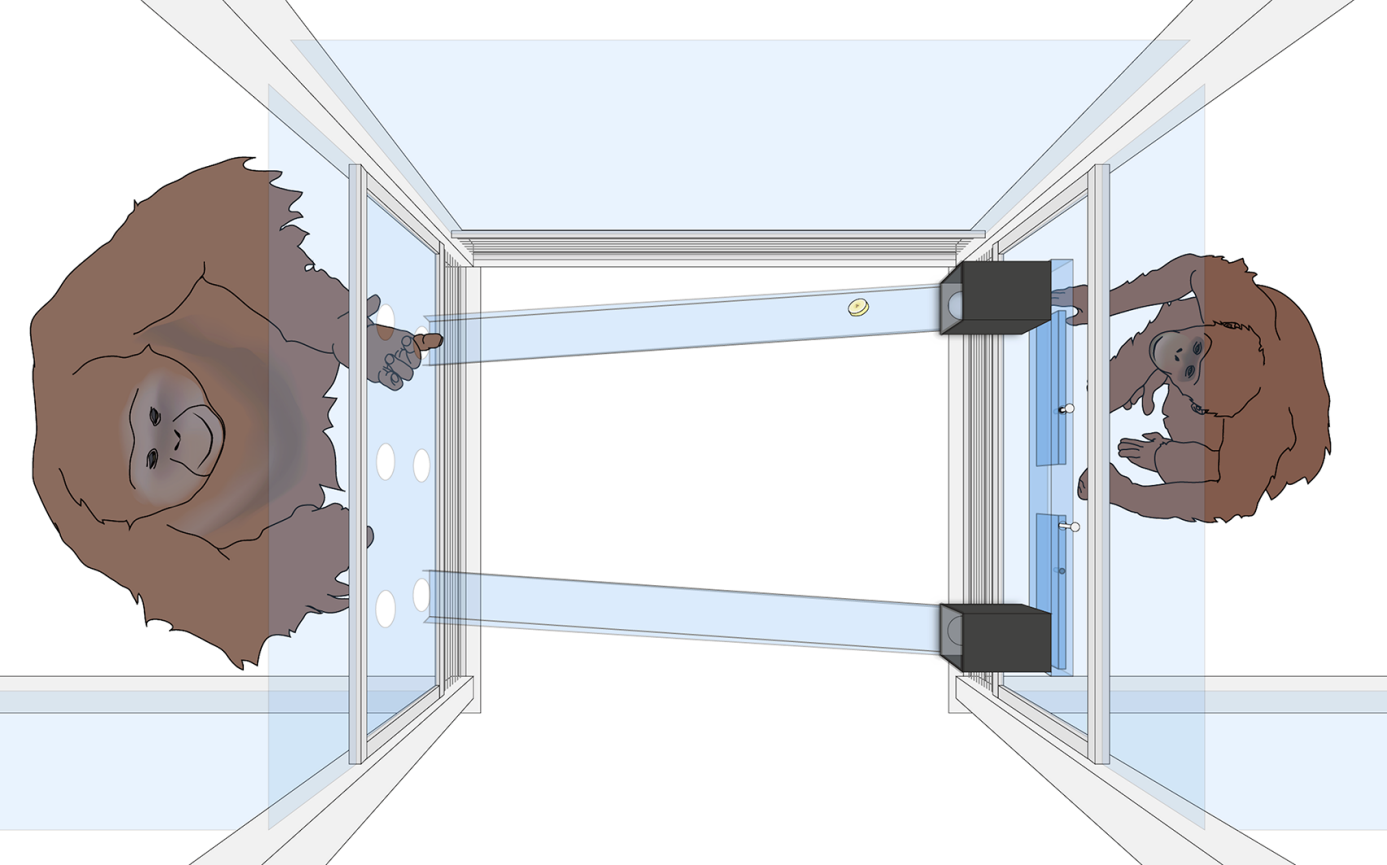
An Illustration of the Equipment Used in the Communication Game. The communicator on the left could see but not obtain the banana pellet hidden in one of the black boxes. The donor on the right could release the food to the communicator, but could not see its location for herself. However, the communicator could potentially indicate the location of the food by pointing through either the top or bottom row of holes on the opposite panel. A locking mechanism prevented the donor from releasing more than one side per trial.

Subjects were tested at the WKPRC in Leipzig, in a pair of adjacent test rooms (2.3m x 2.95m x 3.2m; 2.75m x 2.95m x 3.2m) situated on either side of a 1.1m x 0.95m testing booth through which they could interact. A piece of apparatus designed for the playing of a simple coordination game was placed in this testing booth (see Fig 1).

On one side of the apparatus (the side of the ‘donor’) were two opaque boxes with a transparent front. These boxes, placed about 80cm apart, could be baited and locked. The contents of the two boxes could be seen by the subject sitting opposite them (the ‘communicator’), but not by the donor. At the base of each box, a slide ran from the donor’s side of the booth, to the communicator’s side. This slide allowed the donor to release material from either box to the communicator, by pushing materials down on to the slides, causing them to roll to the opposite side of the booth. The slides ended above receiving trays from which the communicator could retrieve any donated objects.

To release material from the boxes, the donating individual needed to follow a two-step process. The contents of each box could be released by sliding forwards a tray placed inside each opaque box. This caused any material inside the box to drop onto the slide below. However, access to these sliding trays first needed to be gained by moving aside a bar-lock that prevented access to the trays. Sliding the bar-lock towards the centre of the panel both permitted access to one box, and triggered a lock that prevented access to the other box until the mechanism had been reset. Thus only one box could be released at a time.

The equipment was designed so that when food was hidden inside one of the boxes, it could be seen but not reached by the communicator. The donor could not see the food but could release it to the communicator–providing that the communicator was able to indicate to her on which side of the box the food had been hidden. If the donor understood the communicator’s message–and if she felt inclined to respond pro-socially–the communicator could get the food. The walls and panels separating the apes were made of glass and Perspex. Vocal and gestural communication between the two was therefore possible. In particular, the Perspex panel opposite the opaque boxes contained two rows of (6cm diameter) holes through which the communicator could point to the location of the food. One of the rows was placed at the same height as the boxes containing the hidden food. A second row was placed at the same height as the receiving trays in which dropped objects landed. Each row contained three holes: on the left and right (directly opposite both the boxes and the base of the chutes) and in the centre (see Fig 1).

#### Training

So that apes would understand the equipment, all were allowed to explore and interact with it individually, in ten-minute training sessions in which an experimenter continually replenished food in the equipment. Test room doors were left open, so that individuals could move freely between the test rooms on either side of the panel. When the equipment was baited, apes could see where the food was hidden by entering the communicator’s room. Then, by walking into the donor’s room, they could release this food themselves. All tested apes learned how to use the equipment easily, and mastered its use within one session. Additionally, after the conclusion of Study 1 all apes were retested on their understanding of the equipment in a similar setup. All showed no hesitation in successfully retrieving food for themselves.

### Procedure

Two orang-utans played the roles of communicator and donor. The communicator’s role was always played by Bimbo. The donor’s role was played by one of five different females or—in a control condition—by E1. Each female was tested in six sets of three trials, giving 18 trials per female in total and 90 trials overall. E1 played the donor’s role in 30 trials.

Between each trial both ape participants would leave the test room while the equipment was reset. Females were rewarded non-differentially, in the form of food (banana slices and banana pellets) placed in the donor’s test room between each trial. Bimbo was allowed to eat whatever food he could earn by pointing for his partner (females in the test condition; E1 in the control condition). When points in successive trials were not met with successful responses, he was also rewarded on an ad hoc basis between trials, to prevent him from becoming frustrated.

Between trials, one of the two opaque boxes was baited with a banana pellet. Sides were baited in semi-random order (determined using a random number generator, with the same side being baited no more than twice consecutively). Following the baiting of one box, participants were released into the test rooms–first Bimbo on the communicator’s side, and then the female subject on the donor’s side. Each trial lasted for three minutes, starting from the closing of the entrance to the donor’s test room. During this period, interactions between participating subjects were observed and filmed. The trial ended either when the donor (or her dependent offspring) released the mechanism, or after three minutes had elapsed.

#### Control condition

The control condition, in which E1 assumed the role of donor, was run prior to any set of three trials conducted between apes. The purpose of this condition was twofold. First, it enabled us to compare the frequency and accuracy of Bimbo’s pointing for human and non-human subjects. Second, it ensured that Bimbo received a steady supply of food from E1, and remained motivated to engage with the testing in the face of any frustration with female responses (or lack thereof).

In this condition, E1 responded to any points that Bimbo produced by immediately and fully releasing the apparatus on the side to which he pointed, thereby rewarding him for accurate points.

### Scoring

Apes’ interactions were observed to see (1) whether (and with what accuracy) Bimbo would try to indicate to the female (or to E1) the location of the hidden pellet, and (2) whether (and with what accuracy) the females would respond to Bimbo’s gestures by releasing the food to him. Bimbo was counted as producing a point when he inserted one of his fingers into the specially made holes in the Perspex panel on his side of the apparatus. Only points produced after the start of the trial were coded. A point was coded as accurate when it was made on the side of the equipment consistent with the baited box, and irrespective of whether it was produced on the upper or lower row of holes. If Bimbo pointed through than more than one hole at a time or through the central hole, his point was coded as inaccurate.

For responses to points to be included in the dataset analysing comprehension, they had to be (i) produced following a gesture that the donor could see, (ii) produced either during or within 10s of the end of a point, and (iii) constitute a ‘full release’.

#### (i) Visible gestures

Since a pre-requisite of gesture comprehension is the recipient’s being able to see her interlocutor’s gesture, our analyses of pointing comprehension included responses only if donors had been able to see Bimbo’s gestures when he pointed. Because apes’ attention can be fleeting and is difficult to evaluate precisely, we coded this generously. Apes were coded as unable to see a gesture only if they had their back turned to Bimbo for the whole duration of the point. In the vast majority of cases, Bimbo pointed only when others could see his points. In only four trials did he seem to produce ‘optimistic’ points, when subjects could not see his points. In none of these cases was a response produced inside 10s.

#### (ii) 10s time limit

Female responses were counted only if they initiated a full release during or within 10s of the end of a pointing episode. The 10s cut-off point was chosen in order to ensure that subjects’ releasing the apparatus was a response to Bimbo’s points, and to ensure that when individuals released after the end of Bimbo’s point, they were likely to remember the target of his point. This rule was intended to exclude cases where individuals released the equipment long after Bimbo had finished pointing (perhaps because they were bored or had poor inhibition control), and where they no longer remembered the side to which he had pointed. Responses made after 10s were therefore counted as ‘no response’. (This practice follows established procedures in gesture comprehension paradigms involving young children [26] [27].) A total of 19 responses were made by apes within the 10s. (Had a more generous 20s response time been adopted, only one more trial would have been included.)

#### (iii) Full release

Female participants were counted as responding to Bimbo when they both slid open the bar-lock and pushed forward a releasing tray (thereby potentially releasing to him a pellet). The full release rule was introduced because when subjects (and particularly dependent offspring) climbed on the equipment, they sometimes slid open the bar-lock inadvertently. Including the full release rule ensured that trials in which subjects did not make an effort to release any pellet to Bimbo, by pushing forward the tray that (potentially) contained the pellet, were not included as responses in the dataset. Where subjects released incompletely, they were coded as having made ‘no response’ to a point. In Study 1, an adult subject (Padana) performed an incomplete release on only one occasion.

#### Response and response accuracy

Where females released in full within 10s of the end of a point, they were counted as having responded to that point. Additionally, these responses were coded as either accurate or inaccurate. Responses were counted as accurate when the recipient of the point slid open the gate on the side to which Bimbo pointed, and subsequently pushed forward the receiving tray, causing the pellet to fall to him. In cases where the ape pushed the correct tray but the pellet did not fall (for example, because it got stuck in the apparatus), this was nonetheless counted as an accurate response (and Bimbo was rewarded with the retrieved pellet). Responses were counted as inaccurate when the ape slid open and released the mechanism on the side to which Bimbo had not pointed.

#### Releases by dependent offspring

While, in principle, dependent offspring could have responded to Bimbo’s points, in fact none produced any responses that satisfied criteria for inclusion in the dataset.

### Reliability coding

22% of all trials between apes (4 trials per ape subject) were coded by an independent coder, who was ignorant of the hypotheses being tested. She was asked to code the following: (1) Did Bimbo point (yes/no)? (2) Was his point accurate (yes/no/n/a)? (That is: did he point to the side on which the pellet had been hidden?) (3) Was the intended recipient able to see his point (yes/no/n/a)? (4) Did the subject respond within 10s (yes/no/n/a)? and (5) Was the subject’s response accurate (yes/no/n/a)? (That is: did the donor release the equipment on the side to which Bimbo had pointed?) Agreement was 100% (k = 1.00) in (1), (2) and (3) and 95% in (4) (k = 0.93) and (5) (k = 0.91).

Additionally, 20% of trials in which Bimbo pointed for E1 were also subject to reliability coding (6 trials in total). The coder was asked (1) whether or not Bimbo pointed for E1 (yes/no), and (2) whether or not his points were accurate (yes/no). Agreement in both cases was 100%.

#### Dropped trials

One trial (out of 90) was dropped from the peer-peer condition in Study 1, because the subject was deemed to have been in the process of releasing the equipment before Bimbo pointed. As such, this release behaviour could neither be counted as a response to Bimbo’s point, nor as ‘no response’. The final sample was therefore drawn from 89 trials. Additionally, one trial (out of 30) was dropped from the control condition, due to a failure of recording equipment.

### Results

#### Production

Bimbo pointed in 54% of trials for peers (over 89 trials), and in 100% of (29) trials for E1. There was an effect of species on pointing likelihood within a trial (full-null model comparison: *χ* ^2^ = 17.27, df = 1, p = <0.001). Additionally, Bimbo pointed highly and equally accurately for both species (94% accuracy with peers, and 93% for E1; Fisher’s exact test comparing accuracy across conditions, *p* = 1).

Bimbo produced a second point in three trials, each time after his first point was ignored or not seen. Since none of these further points elicited a response, these trials are counted only once each in the analysis of comprehension data. The mean length of his points for females was 14.1 seconds (median 9s; mode 9s; range 1-60s). Since E1 responded to Bimbo’s points immediately, the duration of points produced in the control condition was not calculated.

A full account of production and responses in each trial can be found in the Supporting Information file S1 Dataset.

#### Comprehension

**Fig 2:**
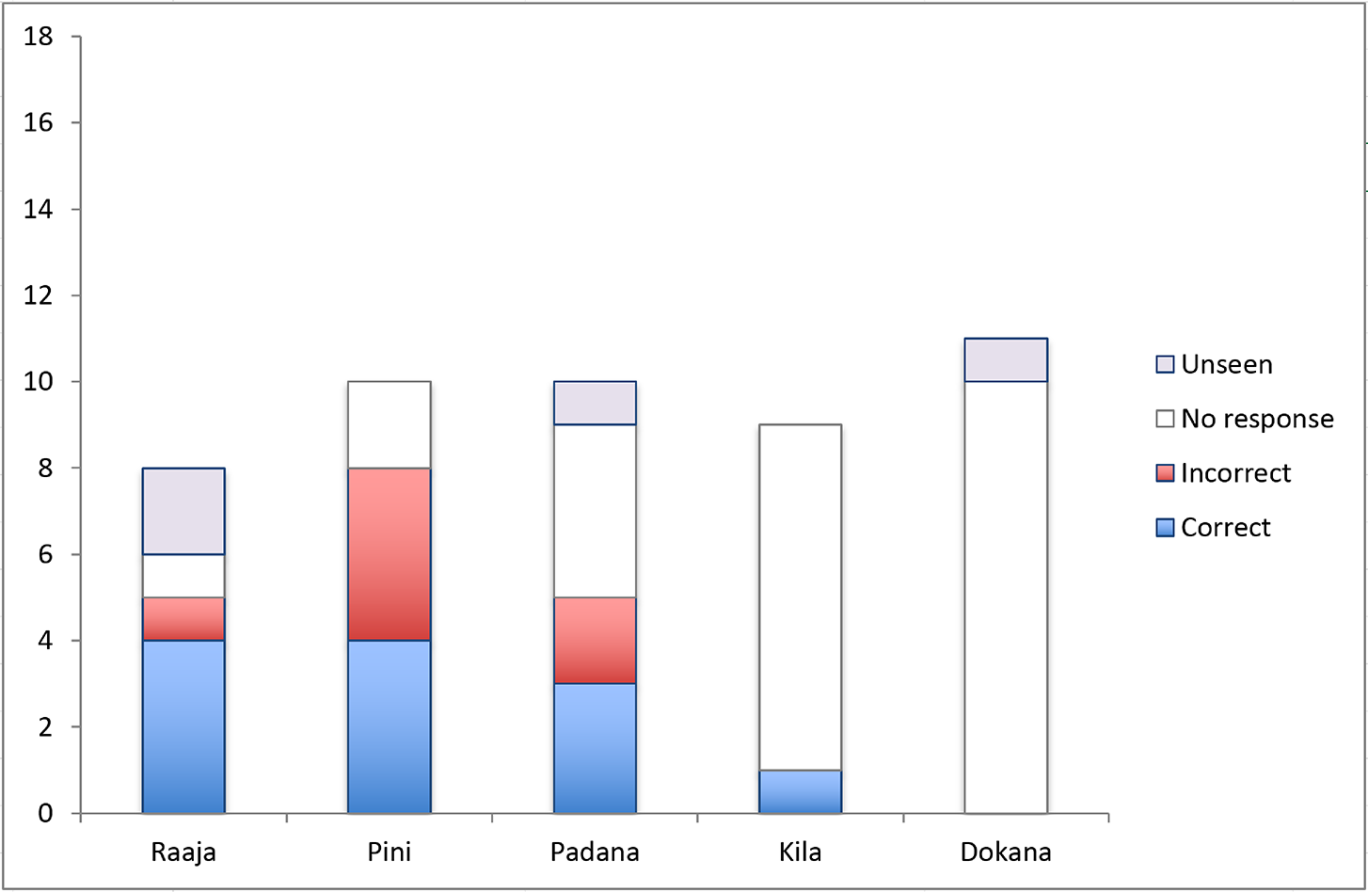
Accuracy of Responses to Bimbo’s Points by Females over 18 Trials. Where Bimbo’s points could be seen by conspecifics, they received one of three possible responses: no response, an accurate response, or an inaccurate response. No female responded inaccurately numerically more often than accurately. However, due to the small sample-size, no statistical analysis is made.

Of the 48/89 trials in which Bimbo gestured for conspecifics, 30 elicited no response from peers. Nonetheless, the vast majority of these points were produced when they could be seen. Attention coding identified only 4/48 points as unlikely to have been seen by subjects or their offspring. These ‘hopeful’ points were included in the dataset measuring Bimbo’s production–but not in the dataset measuring females’ responsiveness and comprehension. Of the 19/44 points that elicited responses, 12 of these were accurate and the remaining 7 were inaccurate (see Fig 2 and Table 1 for distributions).

**Table 1:** Study 1: Individuals’ Responsiveness to and Comprehension of Conspecific Points.

Individual	Responses to Seen Points	% Responsiveness	Correct Responses	Incorrect Responses	% Response Accuracy
**Raaja**	5/6	83	4	1	80
**Pini**	8/10	80	4	4	50
**Padana**	5/9	56	3	2	60
**Kila**	1/9	11	1	0	100
**Dokana**	0/10	0	0	0	n/a
**Combined**	19/44	43	12	7	63

Due to the relatively small sample size, informative statistical analysis of comprehension could not be run–since only Pini generated the six data points required to make statistical significance using a binomial analysis possible, and since we lacked the minimum number of subjects needed for a Wilcoxon signed rank test. Attempts to collect more data were thwarted when Bimbo became frustrated with the test set-up, and refused to approach the coordination apparatus in further trials.

While Pini, Padana, and Raaja responded to Bimbo on more than half of the occasions on which he pointed, he was ignored by Kila and Dokana–giving an overall response rate of 43%. No female responded with less than 50% accuracy.

### Discussion

Bimbo pointed accurately for both orang-utan peers and for the human experimenter. However, he pointed more often for the human experimenter than for his conspecifics. This is likely to be because E1 was attentive and responsive in all trials. By contrast, orang-utans conspecifics often spent little time at the apparatus, depriving Bimbo of the opportunity to point for them. While three females (Pini, Padana, and Raaja) responded to over half of the (visible) points that Bimbo produced for them, Kila and Dokana were almost totally unresponsive–meaning that the overall response rate to Bimbo’s points was just 43%. This lack of responsiveness sometimes led Bimbo to display signs of frustration–including sulking, which he did by turning his back on the apparatus and facing the wall, and (on one occasion) hitting out at the apparatus.

The low response rate here, combined with the low rate of production, compares poorly with a recent pair of studies of peer-peer communication between chimpanzees [28] [29]. However, in both of these studies, the ape in the ‘donor’ role was held in a much smaller test room, with fewer opportunities for distraction. This experimental setup likely facilitated communicative interaction between the conspecifics–because the lack of distractions in the donor’s test room both made it easier for the communicator to gesture for the donor, and meant that the donor had little better to do than attend to and respond to its peer’s gestures.

While statistical analysis of pointing comprehension across individuals was not possible due to the small sample size, three of the four females who responded at all (Raaja, Padana and Kila), responded accurately on numerically more occasions than they responded inaccurately, and Pini responded correctly and incorrectly equally. The overall success rate of 63% is unspectacular. However, it is consistent with meta-analyses of ape performance in other pointing comprehension tasks (see [30]), which suggest that although apes do not excel at pointing comprehension, they tend to be right numerically more often than they are wrong. This suggests that they are at least in some respects sensitive to the information being provided by the pointer.

The ‘points’ that Bimbo produced differed in two respects from characteristically human points. First, all of his gestures were produced on the bottom row of holes on the apparatus (see Fig 1). In other words, he produced his gestures not by holding his hand at the same height as the location of the hidden food, but by placing it at the location where any released food would drop. This may be because when he sat on the floor, this gesture was more comfortable for him. Arguably, though, his ‘points’ were not really points at all, so much as directed (and thereby still referential) ‘begging’ gestures. Such gestures may have been more or less difficult to comprehend than points produced opposite the hiding place of the food.

A second unusual feature of Bimbo’s gesturing was that, while humans and apes alike tend to make direct, ‘ostensive’ eye contact with the intended recipients of their gestures [31], Bimbo did not. Although he looked in the direction of females for whom he pointed, he typically did so with a somewhat averted gaze. While a human might also call out to their partner to solicit their attention, Bimbo remained silent both while pointing and when his points were ignored. This may have had two effects. First, since the use of ostensive gaze and name-calling have been argued to facilitate the interpretation of communicative intentions in humans [32] [33], Bimbo’s behaviour may have increased the chances of females responding to his points inaccurately. Second, his failure to do more to solicit the attention of his interlocutors might have made him comparatively easy to ignore, leading to lower response rates.

## Study 2

In a follow-up study, we sought to further investigate the questions that remained open from Study 1. First, we sought to gather more data about orang-utans’ ability to respond accurately to points by having a human experimenter (E1) play the communicator’s role in every trial. Second, in order to ascertain whether females’ performance in Study 1 could be attributed to the idiosyncrasies of Bimbo’s behaviour, E1 produced points in three different conditions that corresponded to and corrected for Bimbo’s behaviour in different ways.

### Materials and Methods

#### Participants

Participants in Study 2 were the same as those in Study 1, although Bimbo was now tested in the donor and not the communicator role. As before, each of the adult females was tested in the company of a dependent offspring. Only responses by adult female subjects and Bimbo were included in the dataset. This is because E1’s calls were directed only to these individuals; and because dependent offspring could not be tested systematically, since their access to the testing apparatus was dependent on their caregiver’s tolerating their presence at the apparatus. As before, all subjects participated voluntarily. They were rewarded non-differentially for their participation, with food that was delivered on schedule, and water that was available ad libitum. (For further details see the Ethics Statement above.)

#### Equipment and setup

The equipment and experimental setup in Study 2 were identical to Study 1, with the exception that E1 now played the communicator’s role. Additionally, three different styles of pointing were produced in different conditions, to see if this influenced donor responsiveness and comprehension.

#### Condition 1: ostensive directed begging

Condition 1 was intended to resemble Bimbo’s behaviour in the manner of the gestures that were used, but with a greater use of ‘ostensive’ communication–in the form of eye contact and name-calling. E1 produced his points in the bottom row of holes, opposite the hidden pellet, in the manner that Bimbo had done. However, he also retained ostensive eye contact–in the form of relaxed, friendly, direct gaze–with the donor prior to and during pointing production, and he called the donor’s name every 1–3 seconds during the same period (depending on their attention).

#### Condition 2: ostensive ‘human’ pointing

Condition 2 was intended to resemble typical human pointing behaviour. As in condition 1, E1 made ostensive eye contact with the donor prior to and during the production of the point, and called the donor’s name every 1–3 seconds during the same period. However, this time E1 pointed via the top row of holes, opposite the hidden pellet, in the manner that a human might typically do. In this condition, the receiving tray openings were blocked with sliding doors, so as to rationalise E1’s failure to point in the alternative locations.

#### Condition 3: reduced ostension directed begging

Condition 3 was intended to resemble most closely the gestures produced by Bimbo in Study 1. As in condition 1, E1 produced his pointing gesture in the bottom row of holes, opposite the hidden pellet. However, his gaze and attention soliciting were now modelled on Bimbo’s behaviour. As such, E1 looked towards the donor’s face with a slightly averted gaze, and remained silent throughout.

### Procedure

As in Study 1, apes were tested in sessions of three trials–alternating between conditions on different days. Sides were again baited in semi-random order (determined using a random number generator, and with the same side being baited no more than twice consecutively). Before each trial, a small amount of food was placed on a shelf on the back of the equipment, in order to give apes an incentive to approach the testing apparatus.

After apes were released into the donor’s test room, E1 produced pointing gestures and calls in accordance with the condition. The experimenter endeavoured to produce points only when orang-utan subjects were attentive–and ideally when they were standing or sitting at the base of the apparatus, in order to feed. For this reason, the ‘visible gestures’ criterion adopted in Study 1 was not used in our analyses of apes’ responses to E1’s points.

Points were held for approximately 15s each (mean 15.3s, median 16s, mode 16s, range 2-55s). This figure was used to try to match the mean length of Bimbo’s points in Study 1. If no response was forthcoming in 15 seconds, E1 again sought to gain apes’ attention, and subsequently produced a second point. In the ostensive conditions, he elicited apes’ attention by calling their names and looking towards them. In the reduced ostension condition, gaining apes’ attention consisted only of waiting until they were momentarily attentive, while looking at them with a slightly averted gaze. The trial ended either when the donor (or their offspring) released the mechanism, or 20 seconds after the end of E1’s second point, or after three minutes had elapsed.

### Scoring

Ape participants’ interactions were observed to see whether, and with what accuracy, they responded to E1’s points. Responses were counted as accurate only if the apes being tested released the mechanism in full, and in a timely manner, on the side to which E1 had pointed. As in Study 1, for responses to be included in the dataset, they had to be produced within 10s of the end of E1’s point, and for the mechanism to be released in full. While a total of 186 responses were made by apes within 10s, only 8 more responses would have been included if a more relaxed 20s response time were adopted. We also retained the full release rule from Study 1, in order to exclude cases where apes partially released the mechanism while playing with or climbing on the apparatus. In Study 2, incomplete releases from adult subjects were rare (occurring on <10 occasions).

#### Releases by dependent offspring

For reasons described above, releases by dependent offspring were not included in the dataset for Study 2. Nonetheless, a record of these releases (which occurred in 26 of 324 trials) was kept, and can be found in the Supporting Information file S1 Dataset.

### Reliability coding

In the second study, E1 pointed for ape subjects over 324 trials (18 trials per ape, per condition). 72 of these trials (22% of the total; 4 trials per ape, per condition) were second-coded by an independent coder, who was ignorant of the hypotheses being tested. First, she was asked to code whether apes had released the mechanism in full (by both sliding the lock and pushing forward the tray) either during or within ten seconds of the end of E1’s point. Acceptable responses were ‘yes’ and ‘no’. The coders agreed in 96% of cases (k = 0.91). The coder was additionally asked whether the ape’s response was accurate (yes), inaccurate (no), or whether this answer had no application (n/a–because no timely response was made). Here the independent coders’ judgements coincided in 94% of cases (k = 0.92).

#### Dropped trials

In ten trials (6 ‘successful’ and 4 ‘unsuccessful’ releases) subjects were deemed to have been in the process of releasing the equipment at the time that E1 had initiated a point. Since such cases could not be coded as either a response to E1’s point, or as ‘no response’, they were dropped form the dataset. All ten of these cases were viewed and evaluated by an independent second coder, who agreed that they met reasonable criteria for exclusion. The final sample was therefore drawn from 314 trials.

### Analyses

We tested whether orang-utans as a group responded to E1’s points more accurately than would be predicted by chance, and whether any individual responded to these points more accurately than would be predicted by chance. Additionally, we tested both whether (1) the likelihood of an ape’s responding to E1’s point was predicted by condition, and (2) whether in trials in which an ape did respond, the accuracy of the response was predicted by condition.

### Results

#### Comparisons to chance

For both individuals and the group as a whole, we compared the accuracy of their performance to chance. Group-level performance tended towards significance (2-tailed Wilcoxon signed rank test with a continuity correction applied, *V* = 20, *p* = 0.058). However, this *p* value is approximate, since the existence of ties in the data mean that an exact ranking of individual performance could not be completed.

**Fig 3:**
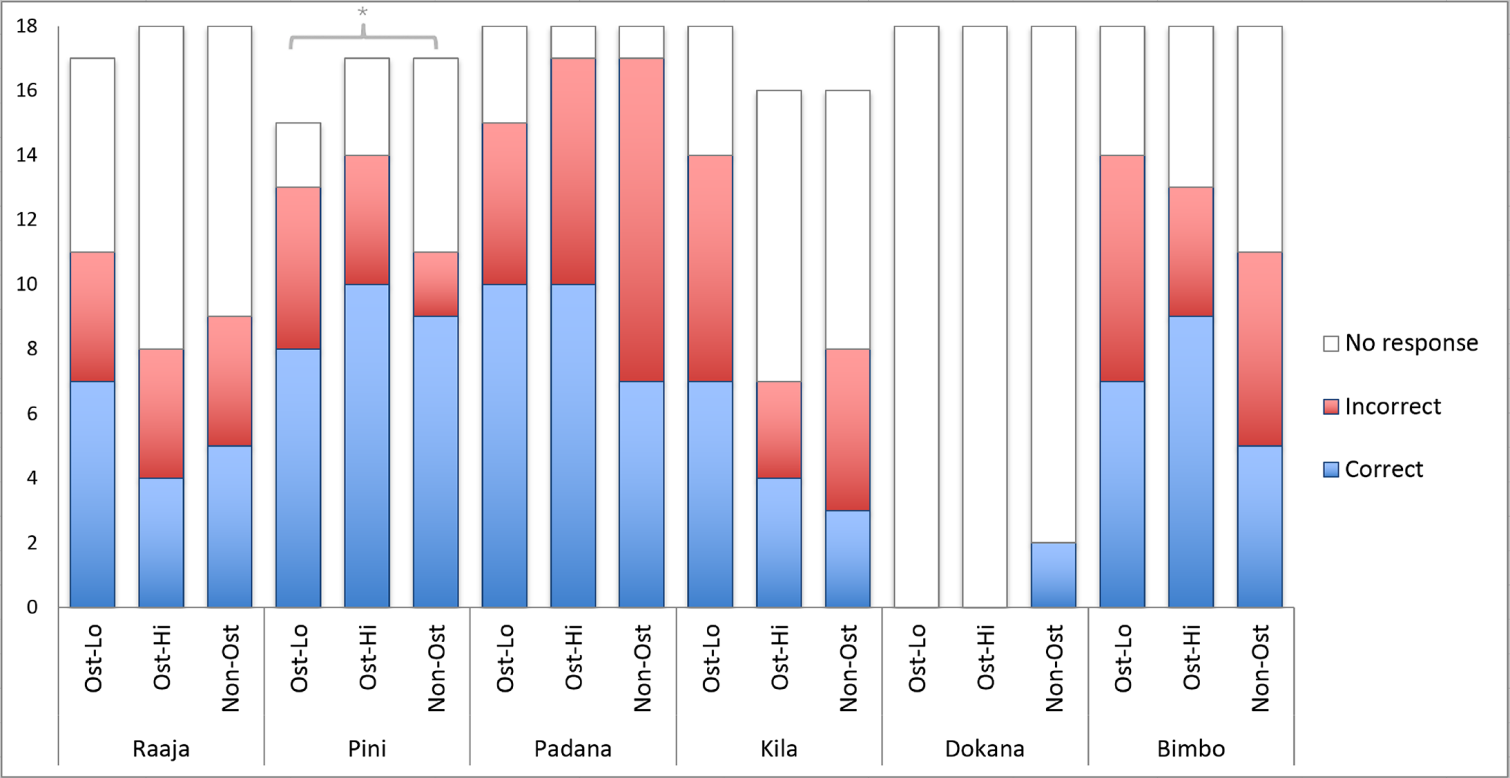
Study 2: Comprehension of E1’s Points in Three Conditions (18 Trials per Condition). No effect of condition was found on either frequency or accuracy of responses. While only Pini was above chance in responding accurately to E1’s points (*p* = 0.014), the group’s overall performance approached significance (*p* = 0.058).

We also tested individual comprehension against chance using binomial tests. Since there was no effect of condition on performance (see following section), and to minimise issues of multiple testing, the different conditions were collapsed within each subject. In this analysis, only Pini (*p* = 0.014) was statistically above chance. Table 2 gives a list of *p* values per individual. Fig 3 shows individual performance across conditions.

**Table 2:** Study 2: Individuals’ Responsiveness to and Comprehension of E1’s Points.

Individual	Total Responses	% Responsiveness	Correct Responses	Incorrect Responses	% Response Accuracy	Performance
**Raaja**	28/53	53	16	12	57	0.572
**Pini**	38/49	78	27	11	71	0.014*
**Padana**	49/54	91	27	22	55	0.568
**Kila**	29/50	58	14	15	48	1
**Dokana**	2/54	4	2	0	100	0.5
**Bimbo**	38/54	70	21	17	55	0.627
**Combined**	184/314	59	107	77	58	0.058**

With the exception of Dokana, all individuals responded to E1’s points on at least 50% of trials (collapsed across conditions). While overall the accuracy of apes’ performance tended towards significance (*p* = 0.058), only Pini responded to these points with greater accuracy than would be predicted by chance (*p* = 0.014). (For full details of individual performance see the Supporting Information file S1 Dataset.)

#### Full-null model comparisons

To test whether there was an effect of condition on either (1) the likelihood of apes responding to points, or (2) the accuracy of their responses, we ran Generalised Linear Mixed Models (GLMMs) with binomial error structure comparing full and null models [34]. (Given the binary nature of the accuracy response, a GLMM is better equipped than an ANOVA to analyse the non-normally distributed data [35].) In both comparisons the same predictor variables were used. The full model comprised ostension, height of point, trial, and sex and age as fixed effects. Additionally, we included the random effect of subject and random slopes of trial, ostension, and height of point within subject, to allow the effect of these predictors to differ between subjects. The model comprising these predictors was compared to a null model lacking ostension and point height.

In the model comparing likelihood of response, there was no effect of condition upon likelihood (*χ* ^2^ (1, *N* = 314) = 3.48, p = 0.18). In the model comparing accuracy of response, which was drawn from a reduced dataset consisting only of trials in which apes responded, there was also no significant difference between the full and null models (*χ* ^2^ (2, *N* = 184) = 0.97, p = 0.62). That is, there was no effect of condition upon either the frequency or the accuracy of apes’ responses.

For both of the models we assessed assumptions of absence of multi-collinearity by calculating variance inflation factors (VIFs), using the vif function of the R-Package. The maximum VIF for the model testing whether or not apes responded to the point was 2.322, indicating no evidence of collinearity. The maximum VIF for the model testing response accuracy was 3.065, showing no strong evidence of collinearity.

We also checked for model stability by excluding female subjects one by one from the data set and rerunning the models to check for variation in the estimates. In the model testing whether or not subjects’ tendency to respond was predicted by condition, Dokana was found to be potentially influential with respect to sex and age. Given her non-participation, this is not surprising. However, with respect to the predictor variables of interest, the model was stable (that is, since she was equally unresponsive across conditions, her contribution did not alter our findings). In the model testing accuracy of response as a function of condition, Padana’s contribution was found to be potentially influential. However, since the full-null comparison remained non-significant even when she was removed from the dataset, the interpretation of our results was not undermined.

We also tested to see if participants’ performance changed over time. We controlled for the possibility that individuals’ performance changed differently over time by including the random slope of trial within the random effect of subject. As before, we tested both the likelihood of their responding, and the accuracy of their responses. We did this by comparing full models comprising trial number, sex, and age as fixed effects. (Given the absence of any effect of condition, trial number was collapsed across conditions–giving a total of 54 trials per subject.) The model comprising these predictors was compared to a null model lacking trial number and the random slope of trial within subject. In the model testing responsiveness as an effect of trial, there was a significant difference between the full and null models (*χ* ^2^ (2, N = 314) = 9.279, *p* = 0.010). To find out the nature of this effect, we created a reduced model without the random slope and compared this to the full model. There was no significant difference between these models (*χ* ^2^ = 0, df = 1, *p* = 0.999), suggesting that the effect did not differ between individuals. A new reduced model dropping the fixed effect was subsequently compared with the full model. This was significant (*χ* ^2^ = 5.749, DF = 1, *p* = 0.017), suggesting that there was an overall effect of trial. The effect was negative: as the test progressed, the likelihood of subjects’ responding decreased.

In the model testing accuracy of response over time, we created–as before–a reduced dataset consisting only of trials where apes responded (N = 184 trials). We then compared the full and null models previously described. In this comparison, there was no difference between full and null models (*χ* ^2^ = 0.182, DF = 2, *p* = 0.913), indicating that the accuracy of apes’ responses did not change over trials.

As before, we assessed assumptions of absence of multi-collinearity by calculating variance inflation factors of these models. The maximum VIF for the model testing change in responsiveness over trials was 2.321, indicating no evidence of collinearity. The maximum VIF for the model testing whether or not apes’ responses changed in accuracy over trial was 3.050, showing no strong evidence of collinearity. We also checked for model stability. In the model testing responsiveness, with respect to the feature of responsiveness the model was highly stable. In the model testing accuracy of response, the model was also found to be stable.

## Discussion

As a group, orang-utans’ comprehension performance tended towards significance. Nonetheless, individual performance was unexceptional: only Pini performed above chance in the accuracy of her responses to E1’s points.

Overall there was no effect of condition on either the frequency with which apes responded to E1’s points, or the accuracy with which they did so. That is, neither the level of ostension nor the height of E1’s points played a significant role in either the frequency or accuracy with which apes responded. Furthermore, while apes’ responsiveness decreased as the number of trials increased, the accuracy of their responses did not change. That is: they lost interest in the study over time, but their responses became neither more nor less accurate.

The small effect of comprehension across subjects is comparable to what has been observed in other studies (see [30]). The finding that only one ape was successful at responding accurately to points is also in line with previous findings, which show that apes are generally unexceptional although not totally lacking in pointing comprehension ability.

Why exactly individuals performed unspectacularly is difficult to diagnose. In particular, we cannot state with certainty that the individuals could not make the required inferences about E1’s communicative goals. Another possibility is that the apes understood E1’s points but were not motivated to respond to them since–by doing so–they would be rewarding E1 with food that they would not themselves get.

Since responding accurately and inaccurately were equally costly options–because apes’ performance was non-differentially rewarded–apes did not stand to lose out by releasing accurately. Furthermore, since apes were not more likely to release incorrectly, and since their tendency to release inaccurately did not increase as the number of trials increased (which might be predicted if they did comprehend E1’s points, but grew frustrated by their lack of reward), there is no evidence that apes grasped E1’s communicative goal, but chose to respond spitefully. Nonetheless, it may be that apes’ performance was undermined by their lack of motivation to reward E1, given that they did not stand to benefit from doing so themselves; and that with greater motivation (in the form of differential food rewards), their performance would have improved.

Given the general lack of accurate responses in all individuals beside Pini, the lack of an effect of condition should also be interpreted somewhat cautiously. For example, the findings do not show that orang-utans are insensitive to ostensive cues. In an easier task, perhaps using a gesture that apes find easier to interpret–like a begging gesture–the presence or absence of ostensive cues might still lead apes to respond to a gesture in different ways.

Some have hypothesised that in humans [33] [36] (and also in domesticated dogs [37]) sensitivity to ostensive cues represents an adaptation that alerts individuals to the fact that they are being addressed with communicative intent. However, particularly in this experimental setup, there is no reason to doubt that apes were generally receptive to the fact that E1 was acting with communicative intent, since (with the exception of Dokana) all were generally responsive and willing to release food to the experimenter. Moreover, given that the apes were generally familiar with similar test-situations, and that in such situations human experimenters often attempt to communicate with them, it’s possible that even in the non-ostensive condition, familiarity with similar test set-ups lead apes to expect communication. In similar experimental set-ups human children are also willing to attribute communicative intentions even in the absence of ostensive cues [26] [27].

## General Discussion

In a previous study [25], Pelé and colleagues found that (1) the orang-utans at Leipzig zoo–and Bimbo especially–pointed spontaneously for conspecifics to request tokens, and (2) that these conspecifics responded appropriately to points by accurately handing over the requested tokens. Study 1 confirms the first finding of the previous study: Bimbo pointed spontaneously and regularly for both humans and orang-utans in order to request food. Moreover, he used his point referentially–by directing it accurately to the particular location of the item that he desired. However, his gestures were somewhat different from human points–in that they were produced both less ostensively and lower down than human points might be.

The findings of Study 2 only partially reproduce the earlier finding that orang-utans understand requestive points. Despite previous findings [25], this finding is perhaps not surprising given that apes’ are typically unexceptional at pointing comprehension; and given the greater difficulty of the task reported here. The fact that Bimbo produced points nonetheless can be explained on the grounds that pointing production is cognitively less demanding than pointing comprehension. This is because in pointing comprehension the recipient has to infer her interlocutor’s communicative goal, based on the actions that the interlocutor performs. Since the producer of a point does not have to infer her own communicative goals, pointing production is easier [3]. At the same time, we would suggest that both the overall performance in Study 2, and Pini’s individual performance, shows that pointing comprehension in this task was not totally outside the ken of orang-utans. It may be that with greater practice, or more substantial motivation, performance would have improved.

It may be that orang-utans’ comprehension performance in both studies was facilitated by the fact that points were produced closer to the location of hidden food than to the alternative location. As such, these points provided spatial cues to the location of the food. This is not a weakness of the study, though; rather it points to a fundamental mechanism by which pointing gestures work–namely by making salient particular features of an environment. In order to coordinate successfully, the donor still needed to infer that the communicator’s gesture was being produced because he (either Bimbo or E1) wanted the contents of the nearby container. Had respondents not made this inference, their releasing behaviour would remain unexplained.

The fact that orang-utans performed well in the Pelé at al. paradigm [25] but less well here can be explained in a number of different ways. A first reason is that in this study the pointer’s point was (necessarily) produced much further from its referent than in the previous study, since participants interacted across a ≈1m wide testing booth rather than on either side of a thin mesh fence. This could potentially make the referent of a point harder to discern. However, the presence of the slides in our setup–which ran from the location of the point to the hiding place of the pellet–served as a visual aid to inferring the target of that point. The side to which the point was directed was therefore particularly salient.

A more likely explanation is that what made our task harder was the difficulty of the inference that was required for successful comprehension of the point. In the Pelé at al. study recipients of the point could themselves see what it was to which the pointer was pointing; and, moreover, they had prior experience of the pointer’s preference for the object to which Bimbo was pointing. Contexts like this are pervasive in human communication. However, they make for communicative interactions that are comparatively easy to interpret [3]. By contrast, in our paradigm the intended referent of the point could not be seen. Rather, apes had to infer the presence of the target from previous experience of the paradigm, and from the pointer’s gesture. Furthermore, they could not use the visual presence of the referent as information in interpreting the possible content of the speaker’s message. Since it was not visually available to them, its presence could not serve to remind them of the prior preference of the pointing individual. As a result, in this task they needed to both suppose that the pointer was pointing to a pellet that they could not see, and to infer that this point was being produced because the pointer desired the unseen object, and was requesting it. Given these ways in which our task was more difficult than the one tested by Pelé et al. [25], it’s unsurprising that–in line with our original prediction–orang-utans should perform better in the earlier study than they did here.

If this is right, the difficulty that apes had in the paradigm tested here, contrasted with their relative success in the earlier paradigm [25], might also tell us something valuable about the limitations on orang-utans’ ability to comprehend pointing. With the caution that is appropriate for comparing data from two quite different studies, we might therefore interpret the data as revealing valuable insights about the origins of pointing comprehension in phylogeny.

It is has been hypothesised that the ability of our ancestors to point to objects, and for the recipients of points to interpret them, may have been a turning pointing in early hominin evolution. Such interactions would have enabled groups of individuals to coordinate their actions with respect to triangulated objects. Once we recognise that pointing comprehension can require inferences of different difficulty, we can suppose that some points may have occurred earlier in phylogeny than others. In particular, one possibility is that pointing for visually available objects appeared earlier in phylogeny than pointing for visually unavailable objects.

The first points produced by our early hominin ancestors may have been produced in relatively intimate interactions. It is already known, for example, that young apes of all species beg for food from the mouths and hands of dominant and older conspecifics [38]. It is a short step from here to imagine cases in which, in order to avoid confrontation, such individuals might also beg for food that was not held by the dominant individual but on the ground in his or her vicinity, and within that individual’s reach and line of sight. Here gestures would perhaps no longer take the form of hands extended towards the individual, but of hands outstretch towards a triangulated referent–perhaps in conjunction with gaze alternation between the addressee and the desired target. Thus, our early points might have more closely resembled referential begging gestures–like the ones produced by Bimbo–than the points that we commonly produce today. This suggestion is consistent with evidence that apes typically produce points for objects that are closer to them (but still unobtainable), while human infants also point for distal objects [18]; and it is consistent with reports that chimpanzees sometimes ‘point’ to the visible parts of their bodies that they would like conspecifics to groom [12] [39]. Other similar gestures are also present in the chimpanzee repertoire–as when, for example, they extend their hand towards a dominant individual in order to recruit support in a conflict situation, while alternating gaze between the aggressor and the individual whom they hope to recruit [40] [41].

Our ancestors’ ability to use points to coordinate their activities with respect to distal and unseen objects, and perhaps objects the significance of which was less evident to their intended audience, plausibly emerged only later in phylogeny, after they had mastered the use of points in more intimate, and easily interpreted interactions. This hypothesis should be tested in future research, by comparing great apes’ comprehension of points in circumstances of varying difficulty.

## Supporting Information

S1 Dataset Production and Comprehension of Gestures Between Orang-utans: Studies 1 and 2. A complete set of coding sheets for all of the data reported in this article.
(XLSX)
